# Attitudes About Informed Consent: An Exploratory Qualitative Analysis of UK Psychotherapy Trainees

**DOI:** 10.3389/fpsyt.2020.00183

**Published:** 2020-03-13

**Authors:** Charlotte R. Blease, Tim Arnott, John M. Kelley, Gillian Proctor, Tobias Kube, Jens Gaab, Cosima Locher

**Affiliations:** ^1^Beth Israel Deaconess Medical Center, Harvard Medical School, Boston, MA, United States; ^2^School of Psychology, University College Dublin, Dublin, Ireland; ^3^School of Healthcare, University of Leeds, Leeds, United Kingdom; ^4^Department of Psychology, Endicott College, Beverly, MA, United States; ^5^Pain and Psychotherapy Lab, University of Koblenz and Landau, Landau, Germany; ^6^Division of Clinical Psychology and Psychotherapy, Faculty of Psychology, University of Basel, Basel, Switzerland; ^7^School of Psychology, University of Plymouth, Plymouth, United Kingdom

**Keywords:** psychotherapy, ethics—clinical, informed consent, survey, psychotherapy education, psychotherapy research, opinions

## Abstract

**Background:** Ethical informed consent to psychotherapy has recently been the subject of in-depth analysis among healthcare ethicists.

**Objective:** This study aimed to explore counseling and psychotherapy students' views and understanding about informed consent to psychological treatments.

**Methods:** Two focus groups were conducted with a total of 10 students enrolled in a Masters course in counseling and psychotherapy at a British university. Questions concerned participants' understanding of informed consent including judgments about client capacity; the kinds of information that should be disclosed; how consent might be obtained; and their experiences of informed consent, both as a client and as a therapist. Focus groups were audio-recorded, transcribed, and analyzed using qualitative content analysis. Coding was conducted independently by three authors.

**Results:** Comments were classified into three main themes: (1) the reasons and justifications for informed consent; (2) informed consent processes; and (3) the hidden ethics curriculum. Some trainees expressed significant doubts about the importance of informed consent. However, participants also identified the need to establish the clients' voluntariness and their right to be informed about confidentiality issues. In general, the format and processes pertaining to informed consent raised considerable questions and uncertainties. Participants were unsure about rules surrounding client capacity; expressed misgivings about describing treatment techniques; and strikingly, most trainees were skeptical about the clinical relevance of the evidence-base in psychotherapy. Finally, trainees' experiences as clients within obligatory psychotherapy sessions were suggestive of a “hidden ethics curriculum”—referring to the unintended transmission of norms and practices within training that undermine the explicit guidance expressed in formal professional ethics codes. Some students felt coerced into therapy, and some reported not undergoing informed consent processes. Reflecting on work placements, trainees expressed mixed views, with some unclear about who was responsible for informed consent.

**Conclusions:** This qualitative study presents timely information on psychotherapy students' views about informed consent to psychotherapy. Major gaps in students' ethical, conceptual, and procedural knowledge were identified, and comments suggested the influence of a hidden curriculum in shaping norms of practice.

**Implications:** This exploratory study raises important questions about the preparedness of psychotherapy students to fulfill their ethical obligations.

## Introduction

Today the importance of informed consent in counseling and psychotherapy is well-established. From an ethical and legal perspective, practitioners are nowadays expected to furnish potential patients with adequate information about the range, nature, and effectiveness of treatments; their timing and duration; common side effects and unwanted events; and costs (if any). The ethical imperative to respect patient autonomy is codified in the professional policies of major clinical psychology and psychotherapy organizations. For example, Clause 3.10 of the American Psychological Association's (APA) Ethical Principles of Psychologists and Code of Conduct, stipulates that, psychologists should, “[o]btain the informed consent of the individual,” and “Psychologists should seek to promote accuracy, honesty, and truthfulness in the science, teaching, and practice of psychology” (1). Similarly, in the UK, the Ethical Framework of the British Association for Counseling and Psychotherapy (BACP) includes among its core principles, “respect for the client's right to be self-governing,” and states that, “We will work with our clients on the basis of their informed consent and agreement” (2).

Despite ethical obligations to respect client autonomy as expressed in professional ethics codes, the question about what informed consent might mean in the practice of counseling and psychotherapy has only recently been the focus of debate (3–6). Notwithstanding this growing body of work, much less is known about how practitioners and student therapists view informed consent to psychological treatments. So far, the limited number of qualitative and quantitative surveys in the US and UK indicate wide variation among therapists and psychotherapy traditions, when it comes to perceptions of the importance of obtaining informed consent (7–10); in particular, one study suggests that practitioners employing insight-orient approaches—whereby clients are encouraged to understand how their past experiences, feelings, and beliefs may influence their present mental state—are the most skeptical about the significance and practicability of obtaining informed consent from clients (10).

### Rationale and Aims

Despite codified ethical norms regarding informed consent, and despite the fact that ethics education has emerged as an integral component of psychotherapy training (11), to our knowledge no study has investigated the views of psychotherapy trainees' or psychotherapy educators about what should be disclosed to prospective clients, and how consent might be obtained. In this study we chose to focus on the perspective of students to explore their knowledge of informed consent including experiences about how it might work in practice, and their judgments of its value and importance. By focusing on the views of trainees, we aimed to gauge the level of engagement and depth of reflection on their ethics education. Clarifying the preparedness of students to fulfill their ethical obligations as professional psychotherapists was therefore a fundamental objective of this study.

## Methods

### Design

Focus groups were employed for data collection as this method is particularly well-suited to obtaining a variety of diverse perspectives, allowing individuals to “share and compare” their understanding, experiences, and opinions (12). Interactions facilitated by focus groups are also helpful in revealing consensus positions; discordant viewpoints; and the “social realities” of a demographic group (13–15). In the current study, this approach was used to elucidate the links between participants' understanding of their psychotherapy ethics and psychotherapy education; their views about their placements and their work with clients; and their own experiences of being in therapy. Finally, since we are not aware of any quantitative or qualitative surveys on psychotherapy trainees' opinions about, and understanding of, informed consent, this methodology enabled the research team to generate a large amount of preliminary data (16).

### Setting and Participants

The research recruited students [used interchangeably with “trainees” in this paper] enrolled on a part-time 3-year training program, leading to an MA in Psychotherapy and Counseling run by a university in northern England. The program includes compulsory courses in counseling and psychotherapy theories; ethical and cultural issues in psychotherapeutic counseling; and research methods. In addition, compulsory course components include work placements and undergoing personal psychotherapy. The MA program is accredited by the British Association for Counseling and Psychotherapy (BACP) in the UK, and individuals who successfully complete the degree are added to the BACP's register of practitioners.

Focus groups were conducted in an easily accessible meeting room in the city in which the university is located; as recommended by Dilorio, the setting was also selected because it was neutral (17). Participants were recruited from among second- and third-year students because these students had already been taught about psychotherapy ethics, and had undertaken work placements with clients. By the end of their second-year trainees have undertaken an average of 50 h involving direct work with clients, and those at the end of their third year will have completed a total 100 h. All participants had already taken part in a teaching session focused on the issue of informed consent and discussed the issues with respect to their own practice and their own experience of compulsory therapy as a client. Focus groups were advertised using posters and via email. Recruitment took place during February and March 2018 and the focus groups were held in April 2018. Students are expected to work in a wide range of settings including the NHS, third sector (voluntary and community organizations), and private practices.

There is ongoing debate about the recommended number of participants in focus groups; however, most researchers recommend enrolling between 5 and 10 participants per group (18–20). Similarly, while there is no consensus about the optimal number of focus groups in qualitative research, our goal was to hold as many focus group sessions as possible. Following recommendations that homogeneity and familiarity among participants helps to facilitate focus group discussions, we aimed to enroll participants into groups according to their year of study (15, 21, 22).

All study procedures were approved by the Research Ethics Committee at the University of Leeds. Prior to attending the focus groups, participants were furnished with information about the purpose and nature of the research. As part of the informed consent process, participants were reminded that the information they provided was confidential and should not be shared beyond the group; that each participant would remain anonymous in the written report of the research; that they had a right not to answer any questions they felt uncomfortable with; that they could withdraw from the study at any time; and that participation or withdrawal would not affect their training. During transcription and analysis, pseudonyms were used to protect the confidentiality of the participants, in line with the study protocol.

### Conducting the Focus Groups

The facilitator (TA) was a postgraduate psychotherapy student who was known to participants but did not have a direct managerial or supervisory relationship with them. Consistency between groups was maintained by an interview schedule and protocol (see Table 1). TA ensured that all participants fully understood the study and demonstrated their capacity to consent prior to initiating the focus groups. Each focus group was audio-recorded, and lasted 60 min. In total, six pre-defined questions were asked.

**Table 1:** Focus group questions.

Questions^*^	**Allotted time** **(MINS)**
1. **What is your understanding of informed consent in the clinical context of counseling and psychotherapy?** *Follow-up question**:* What does informed consent mean to you as a Counselor & Psychotherapist? *Follow-up question:* What does informed consent mean to you as a client?	10
2. **What are your experiences of informed consent as a therapist?** *Follow-up question**:* What are your experiences of informed consent in therapy as a client?	10
3. **When do clients have capacity to provide informed consent and when do they not?** *Follow-up question**:* Can children in therapy give informed consent? *Follow-up question:* Is the building of capacity to give informed consent a goal in therapy?	10
4. **What – if anything – should a therapist disclose to clients about how therapy works to equip them to make a decision about therapy?** *Follow-up question:* What information is ethically relevant for therapists to disclose to the client? *Follow-up question:* Should therapists describe such factors as the importance of the therapeutic alliance, therapist empathy etc.? *Follow-up question:* Should clients be informed as to the effectiveness or evidence-base for psychotherapy or different versions of psychotherapy?	10
5. **How do you think informed consent should be obtained?** *Follow-up question:* Is consent a one-time thing or is it an ongoing process? Or can it be both of these things? *Follow-up question:* How can therapists ensure that the client consents to what is disclosed?	10
6. **Does anyone have anything they would like to add to the discussion?** *Follow-up question:* How have you found discussing this subject? *Follow-up question:* What, if anything, has challenged you today? **Follow-up question*:* Have any of your views changed?	5

* *Follow-up questions were used, as necessary, to help prompt discussion*.

### Analyzing the Focus Groups

Responses were collated and imported into QCAmap (coUnity Software Development GmbH) for analysis. Since focus groups encompassed questions on participants' knowledge, understanding, and attitudes about informed consent, rather than on sensitive, or personal experiences, we applied standard thematic analysis to interpreting the data. This method is well-suited to extracting superordinate and subordinate themes in interviews and focus groups. Three coders (CB, JMK, and CL) independently coded the data to ensure reliability, and each coder brought complementary expertise to the topic of informed consent to psychotherapy: CB as a healthcare ethicist, JMK as a clinical psychologist and psychotherapist working in the USA, and CL as a clinical psychologist and psychotherapist working in Switzerland. The comment transcripts were initially read 3–4 times by CB, JMK and CL to achieve familiarization with the participant responses. Afterwards, each researcher independently coded the data. whereby Brief descriptive labels (“codes”) were applied to each comment (23). More than a single code was applied if comments had multiple meanings. For the inter-coder agreement, comments and codes were reviewed and compared to explore similarities and differences, and discrepancies were discussed until consensus was reached. This process led to a refinement of themes. First-order codes were then grouped into second-order categories and themes based on the commonality of their meaning to provide a descriptive summary of the responses (24).

## Results

### Overview

There were two focus groups, one with five second-year students, and one with five third-year students. All ten participants were female (age range 22–35 years, with an average age of 28 years. All participants had a first degree: as there are no specific requirements for the MA other than an undergraduate degree, each studied for different subject qualifications. The participation rate was 31% (5/16) for second year students, and 38% (5/13) for third years. Although there were no male participants, recruitment reflected the predominantly female student cohorts in both years: 88% (14/16) female students in year 2, and 85% (11/13) in year 3.

The iterative qualitative content analysis yielded 3 main categories: (1) reasons and justifications for informed consent; (2) informed consent processes, and; (3) the hidden ethics curriculum. Each main category contained up to 5 subordinate themes, which are described below with illustrative comments. Numbers in parentheses indicate individual participants.

### Reasons and Justifications for Informed Consent

#### Skepticism About Informed Consent

Participants' comments expressed varying degrees of skepticism about the value of informed consent with some participants raising significant doubts about its importance. A number of trainees reinforced this cynicism by querying whether clients deemed it necessary; for example:

*Informed consent, um, to me it's nothing*. [P4, Year 2]

*Um, don't really see the need in it in some situations*. [P6, Year 3]

*[Y]ou know what, [the] majority of time they're not bothered… they just want counselling*. [P10, Year 3]

A few participants expressed ambivalence about informed consent processes, construing it as an inconvenience or an obstacle that can interfere with early psychotherapy sessions. Some suggested that informed consent was a formality which presented a practical dilemma or trade-off between establishing a therapeutic alliance with the client early on, and procuring the clients' official agreement to proceed; for example:

*I'm like torn between, do I get this new client who I've never met before into the room and like sit down and just be with them for a bit, and then go back to the whole, the formal stuff or do I get that out of the way…first and think right now I've got the consent… but then it's like stale already*. [P7, Year 3]

However, comments also conveyed the view that informed consent was an essential undertaking that had been reduced to a “tick-box” exercise, as demonstrated by these exchanges:

P5 (Year 2): *It feels like in our culture the written signed document is like the, you know it's the holy grail of consent. If you've got that you're set, you know?…That you'd have that. And it's kind of like a box ticked then, um, and so you, you assume that that's all been taken care of…*

P4 (Year 2):'*Cause does feel, just kind of something we have to do and, I don't know*.

P5 (Year 2): *A check-box almost?*

P4 (Year 2): *Yeah, it really does sometimes… And actually, these are people*.

P5 (Year 2): *It's a stark reminder isn't it?*

#### Protecting Client's Rights

Multiple comments expressed the importance of establishing the voluntariness of the client to participate in therapy, and of determining whether an individual had been coerced into treatment.

*Really gotta stress, it's a voluntary service. It's not a forced service… [A]sking them whether they, um, actually want to receive the therapy themselves—if they're not being like coerced or forced by other people that, that they actually want to be there*. [P1, Year 2]

Building on this theme, some participants voiced the concern that vulnerable individuals—especially children and young people—should have a choice about whether they want to undergo therapy:

*Parents might want'em to come but if that, that young person doesn't want to come in, there's no way that you'll make them come in. And you don't want to do that. They've got to come'cause they want to*. [P10, Year 3]

*[It's] really important especially working with like young people: like do you actually want to receive this help?…‘You're not being forced by your parents?'* [P1, Year 2]

An additional, dominant theme was the importance of disclosing information about confidentiality—in particular, making individuals aware of the limitations of confidentiality, and when therapists are legally obligated to inform authorities about what clients had revealed. Notably, these comments about confidentiality were often couched in a way that prioritized the protection of clients' perceived interests; for example:

*If somebody does start to talk about something that you feel might be a safeguarding issue…we're encouraged to say to them, like, ‘Just so that you are aware: it sounds like you are sharing something that, that might pop up. You know, do you want to continue sharing this with me?' And if then if they say ‘yes' or ‘no' that's, like, informed consent within the session*. [P6, Year 3]

*Part of the…informed consent, [is] that, ‘If this comes up, I will have to do something'. Then, they're already aware'cause… they've given informed consent for the counselling and…they're aware of that process*. [P10, Year 3]

#### Protecting Psychotherapists' Rights

Participants also focused on the rights of therapists—in particular, their safety—although remarks were often brief and short. Comments implied that contracts act as a professional shield for psychotherapists. Implicit in trainees' remarks was the notion that contracts were intrinsically related to informed consent; for example:

*There is a contract so it is just, it's laying down, you know what's gonna happen*. [P6, Year 3]

*They signed something…That protects you*. [P8, Year 3]

The need for contracts was viewed as a weightier concern within private practice settings. As one participant reflected within the discussion:

*It's made me think a lot more, like, when I go to private practice—the things that I would get write down and get them to sign, and the things that would just be verbal*. [P8, Year 3]

### Informed Consent Processes

#### Determinations of Client Capacity

A major theme was client capacity to provide informed consent, with some comments indicating confusion about who should be responsible for making such judgements, as this thinking-aloud exchange illustrates:

P5 (Year 2): *Who judges?*…*Ultimately, it's the person working with them, I guess, so it's us as the therapist to make that call. But it feels like…*

P2 (Year 2): *What an onus!*

P5 (Year 2): *Too much*.

Other comments reflected some basic uncertainties about what constitutes capacity. Some participants raised practical concerns about how determinations might work among vulnerable clients. Interestingly, trainees identified a wide range of potentially vulnerable clients including those under the influence of substances; the young and elderly; and clients who may be “*people pleasers*” [P8, year 3], as well as those who are especially anxious; for example:

*[S]ometimes you'll meet someone and you'll think oh they're really drunk, or they're really, really distressed, they're not absorbing, I suppose other times you can be sitting going through a contract with someone and then think wait a minute are they, are they retaining this? And then it's: what do you do?* [P5, Year 2]

*Er, I found it quite alienating for some young people. They either didn't listen or they couldn't process it or they kind of switched off… and I did feel at some moments that they were signing something that they didn't understand*. [P4, Year 2]

Similarly, many participants indicated general agreement that children and minors have the right to refuse treatment but comments reflected uncertainty about ethical and legal processes surrounding consent for young people:

*I don't really know what, like, the actual guidelines are… But it feels like it's the kinda thing you've just got to use your judgment as you go along like I guess*. [P8, Year 3]

*I don't know the rules about this… So, what if the child, like, would, just want to please their parents, say, like, they're I don't know, in an abusive relationship, and the parents are forcing them to go in there, like, what would you do in that situation, like, what's legal?* [P6, Year 3]

#### Disclosure About What Happens in Sessions

In contrast to voiced misgivings about the value of informed consent among participants, there appeared to be general consensus that prospective clients should be informed about *what* goes on in therapy sessions. Despite this agreement, a number of comments were vague about what disclosures should entail; for example:

*[Informed consent is about] providing the client with as much as you can in that moment, um, information about what, potentially, the therapy could entail or how*. [P4, Year 2]

*It's [about disclosing] what could happen to them, so that when they're agreeing to begin treatment, they're fully aware of what it entails*. [P8, Year 3]

Other comments expressed the need to provide information about how therapists work; indeed, some participants were adamant that clients should be furnished with this information.

*I think it's really important to say how we work*. [P3, Year 2]

*It feels, it feels ethical, it feels like number one priority is that the… person knows what is going on and is in control because otherwise it feels like it undermines for me everything that you're trying to do in counselling*. [P5, Year 2]

Drilling down into more detail, a few trainees felt that information about techniques should be disclosed to potential clients. One participant explicitly urged that clients should be made aware of other treatment modalities to facilitate their choice about how to proceed in therapy:

*I think that's absolutely vital [to disclose how therapists work] because the way one person works, er, is obviously different to another, and, um [clients] may realize that they want something else and you've got to allow them to have that decision*. [P3, Year 2]

*It's important for the client to know…how we practice… Because everyone's got so many different approaches, and clients don't necessarily understand all the different theoretical approaches that we might be coming from…So… if someone goes to a cognitive behavioral therapist and then someone comes to a relational therapist do they understand the differences in how you're going to be working with them or what it means for them?* [P7, Year 3]

In opposition to this view, a number of trainees suggested that providing information about different treatment techniques risked confusing clients:

*I would like to know if I saw any counsellor…what their approach is but that's because I have knowledge of counselling approaches*. [P8, Year 3]

*It's a line isn't it, er, between being transparent and then moving into being like psycho-educational*. [P5, Year 2]

*I'd never impose a technique or some sort of jargon that could again completely go wrong with them because they are so vulnerable… Imposing a language onto someone I guess for me doesn't feel right*. [P4, Year 2]

Finally, when asked whether information about the therapeutic value of common factors—such as practitioner empathy, and therapeutic alliance—should be included in disclosures, participants were overwhelmingly dismissive of the idea. Some trainees suggested that clients would instinctively experience these factors, as therapy unfolded, as illustrated by this comment:

*I don't think I would talk to my client about that. I'd rather, you know, I'd want it to be felt…And for them to have the experience rather than for me to keep educating them on what's happening*. [P5, Year 2]

#### Disclosure About Evidence-Based Information

Strikingly, participants were skeptical about the relevance of evidence-based information to practice. A derisive tone was conspicuous in some comments which conveyed significant doubts about the value of the evidence-base to client disclosures. Here again, some comments also accentuated the risks of perplexing clients; for example:

*I suppose in doing that [disclosing evidence-based information] you then are trusting the evidence base [laughs] as some sort of truth… Look at all these RCTs we've got. [Laughter]. And actually, when you look at the measuring tools you're just like what?! [Laughs] So, yeah*. [P5, Year 2]

*Yeah probably would… want to have an awareness of it myself to be kept up-to-date with what's going on, I guess, but yeah, just really cautious of where the evidence is come from…Is it really meaningful to the client or not? Or are they going to bamboozled with statistics? That could be an anxiety provoking thing for them*. [P2, Year 2]

Relatedly, when asked whether it was important to disclose information about harms and risks, participants tended to be unconvinced, or admitted that this was an issue they had not fully considered; for example:

*You don't need to think seriously about that… I can never picture a scenario where I'm telling my client what [laughter]… harm it can have [laughter]…* [P5, Year 2]

*Makes me realize I haven't really thought about [it]*. [P3, Year 2]

*I think… [the] key bit [is] that you can get worse before you get better. I think I do say that to clients. They're, like, ‘this is awful', and I'm just, like, ‘You [ha], like this is something that you need to know…but yeah… it will not be easy…* [P6, Year 3]

#### How to Disclose Information

Against varying views about *what* should be divulged to clients [see superordinate topic: Disclosure about what happens in sessions], trainees expressed a variety of perspectives on *how* such information should be disclosed. Some comments suggested that disclosure should be verbal, and “*in writing as well*” [P2, year 2]. A recurrent view was that, regardless of the medium, information should be accessible; for example:

*Making it really basic and breaking it down without like really fancy words… Maybe something about working as an integrative therapist, it might be helpful, a little pamphlet or something…to give to the client—‘so take this home—for you to read… it just gives you a bit of information'*. [P6, Year 3]

*[E]qually we have clients who don't read. Can't read and write. Have never learnt… So, it's a mix isn't it I suppose… of what works for one doesn't always work for someone else*. [P2, Year 2]

*I would like going forward I guess my own practice in the future to be open to different methods of communication*. [P4, Year 2]

One student elaborated that the nature of their practice would influence what they would disclose:

*[I]t's just something about private practice for me that… just shits me up…If I was working privately I think it would be good to have a pamphlet and have a bit of information for them to take home*. [P6, Year 3]

Other participants expressed the view that clients would tacitly grasp the nature of therapy only by experiencing it, with some comments indicating that this could substitute for explicit disclosure of information; for example:

*See I don't read through the contract, um, partly because like you said, the… mass of it [laughs]. I just think people kind of glaze [laughs] and they're not absorbing it anyway and it's kind of, I want that first session be more about, um, them having experience of what therapy is*. [P5, Year 2]

*I think it took them a little while to be like, ‘This is actually not what I, kinda, signed up for, it's not what I want. Um, and yeah and I decided well actually, it's not for me', then left, um but yeah maybe, like, takes a few sessions for them to, like, absorb that, like, okay this is what counselling is, ‘I wanna go forward with this.'* [P1, Year 2]

#### Establishing Consent

Similar to responses about how to disclose information, participants endorsed the view that tacit rather than explicit consent from clients was more reasonable; for example:

*Really simply, it's kind of like, the client showed up, out of their own free will to have a session with you, and if the client comes again out of their own free will to have a second session with you…They just won't come back if they don't want to*. [P6, Year 3]

*[I] think there's just an assumption that clients, kind of just, vote with their feet and if they happy to come they'll come*. [P10, Year 3]

Elaborating further, some trainees emphasized that determinations of client consent were a matter of tacit expertise on the part of practitioners:

*It's a felt sense as well—like you intuit things don't you?…They might be saying “yeah yeah, totally” but you can tell, they're kind of like, “Oh we're not comfortable being there”… so it's about using our skills as a therapist*. [P5, Year 2]

*You are going through it but you don't explicitly say to the client like, ‘do you want therapy?'* [P1, Year 2]

Dissenting from this perspective, however, one participant proposed that, “*Looking for some kind of affirmation, some you know, nod and agreeing that they've understood”* [P3, year 2] was necessary. Combining disclosure of information with the issue of obtaining clients' consent, a key question raised by many participants was whether these joint issues should be conceived as discrete, one-off events, or as an ongoing process. Again, some trainees questioned whether the first session was the appropriate time to provide information; and a number of comments revealed that participants were spontaneously deliberating and debating how best to enact appropriate disclosures during the focus group discussions. For example:

*I think for me it comes down to it being more of a process than an event*. [P5, Year 2]

*Revisiting it… perhaps it's a good idea to do that…So I'm thinking about revisiting which I think… is brilliant because that first initial session so much [is] going on, um, but thinking about… how I would learn as well*. [P2, Year 2]

*I think it's being aware of that all the time when, you know always checking out with them*. [P6, Year 3]

*I think as well for me it's… revisiting it, I don't think you should assume… that, I guess, your clients [are] in the same place they were or even remember sometimes*. [P4, Year 2]

### The Hidden Ethics Curriculum

#### Experiences as a Client

Multiple comments underscored the relevance of the so-called “hidden curriculum” in psychotherapy education. The term “hidden curriculum” has its roots in research in medical education (25) and refers to the effects of socialization into medical practice in undermining formally declared training objectives in medical curricula. In this paper we have adopted and extended the use of the term to the domain of psychotherapy ethics, and take it to refer to the unintended transmission of norms and values in training that undermine the explicit guidance, recommendations, or values expressed in professional psychotherapy ethics codes. Personal psychotherapy comprised a compulsory feature of the course for trainees. Presumably students were aware of this mandatory aspect of the course prior to enrolment; in addition, justifications for undergoing therapy were discussed with trainees. Interestingly, however, a few participants considered this obligatory course component to be a violation of their own freedom of choice to engage in the psychotherapy process. For example:

*We are on this course being kinda forced [in]to having therapy. No, I'm not saying that a bad thing er…and it's been really positive for me so it's not that I'm against therapy. But we are being forced into a certain number of sessions as well, and I think that, y'know, do we really have informed consent over that? Or is it something we're being coerced into doing?* [P3, Year 2]

In addition, participants frequently reflected on their own experiences as a client in therapy, recalling how therapists broached issues pertaining to informed consent. Multiple comments suggested that trainees-as-clients had limited experiences of being provided with information about therapy by professionals; for example:

*[I'm] just trying to think… like… with my… two therapists… that I've had. One was really clinical and she was very much, like, I think we did contracting at the beginning but it was like she'd sit at her computer desk and talk to me which was very like power-orientated but then… I didn't really feel the need to ask her about her approach. And then the second one was like a home-based counsellor who again didn't really tell me much about him at all*. [P5, Year 2]

*There wasn't even like an initial session. [It] was just like boom—there we go—straight in*. [P1, Year 2]

A number of the participants indicated that formal aspects of consent—such as signing a contract—were uncommon experiences. Justifying these practices, some participants emphasized that their prior knowledge better equipped them to undergo psychotherapy; for example:

*He wouldn't sign anything or read anything out. Um, but I knew what to expect… I didn't leave thinking ‘oh gosh', you know, but then again, I'm also a trainee therapist so then I kinda know… what to expect*. [P6, Year 3]

However, one participant provided a critical perspective on what she had experienced:

*You [other trainees] knew, you knew something about it, imagine a client who has no clue… Just not having that, that's a bit horrifying*. [P3, Year 2]

Interestingly, however, most comments did not illustrate reflective connections between personal experiences of psychotherapy, and objective evaluations of informed consent processes, or of ethical practice, more generally. For example:

*I've never even thought about informed consent in therapy as a client*. [P10, Year 3]

*Nah I, I think… as a trainee therapist when you [are]… a client then you know… [T]hen if they do something wrong, you're like Ha! [Laughter]. You know [laughs] but otherwise you, yeah, I don't really have any other expectations*. [P9, Year 3]

#### Experiences on Placements

Students' accounts of their placement experiences provided a rich source of first-hand observations and expectations surrounding informed consent practices. Notably, a few participants recounted that disclosure of student status was not standard practice. One student, who felt strongly about this, noted:

*And for me it's about kind of really heavily linked with ethics in, um, I do tell my clients I'm a student. I feel I should do though I know some people don't*. [P3, Year 2]

Some trainees expressed positive impressions of practice experiences; for example, with respect to provision of accessible material about therapy, one participant noted:

*I think the organization I'm with is very on it about making the information as accessible as possible, simple language and different kind of ways of delivering that*. [P5, Year 2]

However, this was not a shared perception; another student recalled:

*I used to do a placement with young people, and actually I spoke to the lead about making the forms accessible because [of] the language*. [P4, Year 2]

A few trainees described sessions with vulnerable clients some of whom they believed lacked capacity to provide consent, or whom they judged to have been coerced into treatment. For example:

*[I] had an elderly client who I only had for three sessions…My placement supervisor just said, ‘I'm not sure counselling is for her'. She was in her 80's. ‘But you go and…see what you what your thoughts are.' And she nodded off quite a lot and through the session and she wanted to be in counselling because of alertness but then it turned out that her daughter volunteered her within the charity, and so when I discussed kind of ending and whether or not counselling was right for her she said, ‘Oh but my daughter's going to kill me'. So, did she fully have informed consent?…I didn't feel she did, and…I felt really quite strongly about that in terms of we do need to end this*. [P3, Year 2]

*[I] was just immediately thinking about a client that I had who, um, came for a first session and her husband wanted to stay in the therapy room and I…thought …This can't happen and… they did the assessments like before they sat with the counsellor, and I was, like, surely this was explained [laugh] in the assessment? Um, and eventually you know he left but she didn't have any medical diagnosis but it, it really felt to me like there was…she was but, older lady and she… didn't have a clue where she was. Didn't know why she was there—she just knew it was good for her'cause her husband had told her she needed it…I didn't even carry on with the session. This is not right*. [P7, Year 3]

Similarly, some students were unclear about who was responsible for providing information to clients, or securing contracts. One participant expressed that she was “*quite surprised*” [P1, year 2] that a contract had already been undertaken with the client, describing it as “*quite nice actually… so I can go straight in*” [P1, year 2]. Another described witnessing divergent procedures at different placements:

*Whereas, I'm wondering now'cause in one placement I do the contract, and in the other placement, um, the contract is already done at an assessment stage so then I just kind of say who I am and how I work and say [do] you understand about confidentiality?…But it's quite informal…It's kind of creating a question for me…* [P3, Year 2]

Seemingly unselfconsciously, however, some participants' comments intimated that their own clients misunderstood the nature of therapy sessions. Such comments suggested that discrepancies in client's expectations were revealed as treatment ensued; for example:

*I've worked for a couple of clients in a new placement where after the first session they've come back and they've been like, ‘Oh, actually that was a lot different from what I was expecting'… I think they were expecting like some sort of diagnosis, more of a psychiatric type thing and took them a few… sessions to be like, ‘actually this isn't what expected at all'*. [P1, Year 2]

## Discussion

### Principal Findings

To our knowledge, this is the first study to explore psychotherapy students' views about informed consent in counseling and psychotherapy. Overall, this exploratory study found that trainees were confused or uncertain about basic conceptual and practical issues relating to disclosure processes, with many students expressing skepticism about the value of informed consent. The candor of some comments, including remarks that suggested that informed consent was an inconvenience, may suggest deficiencies in formal ethical education—shortcomings that may be reinforced by the “hidden curriculum” including poor modeling by personal psychotherapists. However, on an optimistic note, it should be emphasized that participants' comments also indicated both perspicuity and clear expressions of ethical concern—including the critical reflections that informed consent risked being reduced to a “check-box”; worries over vulnerable clients being coerced into treatment; and several observations that informed consent practices in work placements were unclear and/or inconsistent.

Notwithstanding these insights, trainees appeared unfamiliar with elemental aspects of informed consent. While the reasons for this are unclear, these omissions may indicate lack of formal knowledge or training on informed consent, or problematic role modeling in psychotherapy sessions. Although participants clearly had strong attitudes toward the processes pertaining to informed consent, they didn't emphasize guideline-based facts or recommendations. Comments revealed that some trainees used their own discretion about whether to be transparent with clients about their student status, behavior that contravenes ethics guidelines. Clauses 82(a) and 45 of the BACP Ethical Framework stipulate, respectively: “In the interests of openness and honesty with clients: trainees on a practitioner-qualifying course working with clients will inform clients (or ensure that clients have been informed) that they are trainees,” and “Whenever we communicate our qualifications, professional experience and working methods, we will do so accurately and honestly.” Other confusions arose with respect to therapists' responsibilities in determining client capacity to provide informed consent. A number of participants also expressed uncertainty about guidelines and regulations concerning consent for younger clients, even though many trainees appeared to have experience in treating adolescents.

### Paternalism and Respect for Client Autonomy

Similarly, although trainees embraced ethical concerns about the need for clear and accessible disclosures with clients, oftentimes comments suggested lack of knowledge about the need to respect patient autonomy and choice. Nevertheless, participants had a strong internal “moral compass” and had an intuitive sense for the necessity of providing informed consent. Comments indicated a keen awareness that individuals who seek counseling and psychotherapy may be especially anxious, and unable to absorb information, especially in the first session. Participants in our study appeared to interpret such cases as a justification for why informed consent might fail.

This signaled lack of education about the concept of paternalism in health care. The concept of paternalism has been defined as, “the interference of a state or an individual with another person, against their will, and defended or motivated by a claim that the person interfered with will be better off or protected from harm” (26). Strikingly, the word “honest” was used only once during focus group discussions. As psychotherapy ethics literature emphasizes, challenges associated with providing informed consent do not abnegate professional responsibility: rather, adequate information must be provided, and processes adapted to ensure that clients are afforded the moral status of autonomous agents; as Trachsel et al. argue, “informed consent might be slightly more complex than for non-psychotherapeutic treatment, but it is nevertheless a central requirement in ethical terms” (4). Moreover, in health care, and in psychotherapy, as is reflected in ethics codes, strong reasons need to be advanced to defend the violation of an individual's autonomy. In the UK, the Mental Capacity Act of 2005 states that there must be a presumption of capacity, on the part of practitioners, that patients can make treatment decisions, and furthermore, that the burden is on health professionals to justify that an individual lacks reasonable capacity (27).

### Informed Consent as a Process

Encouragingly, and in line with recent literature on psychotherapy ethics, a few trainees described informed consent as best conceived as a process; others appeared unfamiliar with this interpretation—even while welcoming the idea of revisiting disclosures as therapy progressed. However, a number of trainees expressed the belief that clients could only understand therapy by experiencing it—a view that may also be prevalent among professional psychotherapists (9). Opposing this perspective, ethicists acknowledge that informed consent is not simply a one-time event but argue that this does not provide justifiable grounds for omitting adequate, accessible disclosures in early sessions (6, 28, 29); rather, they argue, informed consent to psychotherapy should be understood as an ongoing, bidirectional process that facilitates refined awareness on the part of clients (28, 30).

The question about what to disclose also revealed disparities between trainees' views, and duties as outlined by ethicists. No comments detailed the need to disclose practical information: for example, duration of therapy sessions; expected length of treatment; or the treatment approach. Whether such information is routinely provided by students was unclear from our findings. Although such information may seem obvious to therapists, as Fisher and Oranksy have argued, it is important in securing adequate informed consent (31). Many clients may be unaware of such information, especially those who consult psychotherapists for the first time, a point that was emphasized by a number of participants. Not properly being informed about these issues might also be particularly problematic for clients who are shy or anxious, and reluctant to request basic information. Consequently, lack of adequate information might exacerbate feelings of uncertainty about psychotherapy.

Oversights also arose with disclosures about the nature of therapy sessions. While many trainees agreed that clients should be “*fully aware of what [therapy] entails*”, and that, “*it's really important to say how we work*” for most students these sentiments seemed to stop short of a commitment to divulging specific treatment techniques, or indeed of the range of possible treatments that might be available to clients, and justifications for these omissions adopted a paternalistic stance. It should also be emphasized that the BACP commitments are somewhat vague on this issue [see: Clauses 3b & 4a (2)]. Conceivably, some students or practitioners may be trained to consider that certain modalities or approaches in psychotherapy preclude disclosure of techniques on the grounds that this interferes with the very nature of therapy. In contrast, ethicists urge that psychotherapists have an obligation to inform patients—in clear and understandable language—about the techniques being employed in therapy, including information that a range of other psychotherapy approaches may also be appropriate for clients (4, 6, 32).

### The Evidence-Base in Psychotherapy

Worthy of emphasis were trainees' responses to the “evidence-base” in psychotherapy. Participants were particularly skeptical about psychotherapy research, and of the relevance of communicating “evidence-based” findings to clients. Again, adopting a paternalistic standpoint, some argued that such disclosures could be “anxiety provoking.” Some trainees appeared to conflate evidence-based practice (“EBP”) with a narrower conceptualization of research derived from randomized controlled trials (RCTs)—a point that we argue, is worth reflecting on.

Although research in psychotherapy remains the subject of ongoing debate with much focus on what constitutes appropriate standards of evidence (33–37), we suggest that therapists have a duty to keep abreast of these challenging issues. Taking evidence seriously is an explicit commitment of the BACP, which states that “[Clause (14)]: “We must be competent to deliver the services being offered to at least fundamental professional standards or better”; and “We will keep skills and knowledge up to date…: [14(b)] by keeping ourselves informed of any relevant research and evidence-based guidance.”

The BACP's *Ethical Framework for the Counselling Professions* further stipulates that it values research for “enhancing our professional knowledge and providing an evidence-base for practice in ways that benefit our clients” (Good practice point 68)” (38); and its website emphasizes, “We take a pluralistic approach to research, including data from trials, practice-based studies and qualitative, theory-building cases” (39). In this way, similar to the APA (40), the BACP appears to endorse a “thick” conceptualization of evidence that encompasses a wide variety of basic scientific research into psychotherapy and practice including research on clinical expertise; evidence relating to patients' judgments and preferences; and findings on the effectiveness of treatments, including mechanistic evidence, hypothesizing how treatments work. Notably, this evidence includes—but is not limited to—RCTs, systematic reviews, and meta-analyses aimed at investigating the relative and absolutely efficacy of psychological treatments.

Despite explicit pronouncements of national clinical psychology and psychotherapy organizations to embrace a thicker interpretation of evidence, online repositories of information about evidence-based psychotherapy often appear to place greater weight on a narrower interpretation of evidence resulting from RCTs—that is, of “evidence-supported treatments” (EST) (33, 41). For example, in curated web resources for practitioners, the APA's Society for Clinical Psychology lists “Research-Supported Psychological Treatments” (42); the BACP also provides a list of links of studies primarily aimed at investigating the absolute and relative effectiveness of different version of therapy (or ESTs), rather than listing research on the nature of expertise; meta-theoretical findings; or process research (43). In light of this emphasis on EST, it is perhaps not surprising that many trainees seemed to conflate EBP with this narrower emphasis on RCTs. Therapists who predominantly practice psychodynamic, humanistic, and existential approaches, appear to be most skeptical about the value of psychotherapy research (33, 41). Nonetheless, every psychotherapy tradition depends on some implied standard of evidence; even psychodynamic psychotherapies which employ techniques associated with acquiring insight or resolving inner conflicts make assumptions about what constitutes evidence of insight or resolution, and how improvement might manifest itself (29). These wider considerations about evidence-based practice appeared to have been overlooked by the majority of trainees in our study. Moreover, a common theme was that practitioners' “expert judgments” in intuiting clients' views and their assent could substitute for clients' explicit consent and feedback. These beliefs contrast sharply with evidence that psychotherapists' subjective impressions of patient progress and treatment efficacy are often inaccurate or misleading, and may be prone to self-serving biases (44–46). Trainees appeared to be unacquainted with findings that ongoing feedback from clients improves outcomes (47–51).

A further consideration relating to EBP and informed consent is disclosure about availability of psychotherapy services to prospective clients. For example, if some modalities are not available on the NHS an ethical issue arises in disclosing the range of psychotherapy approaches suitable for an individual's needs if clients may need to pay for some of these services privately. Certainly, the case can be made that prospective clients should be fully informed about their options, regardless; however, we suggest that the BACP, APA and other relevant professional guidelines should provide clear advice to clients and practitioners on best practice pertaining to constraints on choice of psychotherapy relating to costs, access, and (where relevant) insurance coverage.

### Disclosure of “Common Factors”

Related to the question of evidence-based information, participants' views about communicating the value of “common factors” in therapy [e.g., the role of therapists' empathy; and, of the importance of clients getting on board with treatment techniques; etc.] appeared diametrically opposed to recommendations by some ethicists and psychotherapists who argue that disclosing such factors is an ethical imperative (5, 6, 52). Despite ongoing disagreement about the relative importance of specific techniques in psychotherapy research (e.g., of cognitive restructuring techniques in cognitive behavioral therapy, or of insight-techniques in psychodynamic traditions) findings indicate that shared factors across psychotherapy modalities play a significant role in treatment outcomes, leading some prominent psychotherapy researchers to argue that the “common factors” are the major mediators of change (53, 54). Although this claim remains controversial (55, 56), there is compelling agreement across diverse psychotherapy traditions that such factors play a significant role in treating clients (5, 32, 52, 57). Extending this view, ethicists have argued that by placing too much emphasis on particular techniques, and undervaluing common factors, clients may be poorly positioned to take action, especially if they perceive that therapy is not working; in such circumstances, lacking appropriate information, it is conceivable that some clients may drop out of therapy, falsely believing that a particular version of therapy—or indeed, all talking therapies—are “not for me” rather than being cognizant of the potential role of client and therapist factors in outcomes (57). Recent experimental research further challenges the suggestion that clients may intuit the value of common factors, and that communicating such information is redundant (58). Insufficient knowledge about therapist factors can lead to particularly dramatic consequences if clients attribute perceived treatment failure to themselves. Especially in severely afflicted persons, such an interpretation might result in despair (and possibly even suicidal thoughts) if they put all their hope in one therapist and end up thinking, “I am a hopeless case because therapy could not help me.” On the other hand, lack of information about the importance of client factors can be a risk factor of low treatment success if clients are not aware of the necessity of their own active role in the therapy process.

### Harms and Risks of Psychotherapy

A number of trainees were dismissive about the disclosure of possible harms and risks of psychotherapy as well as unwanted events. Resolving what should appropriately be disclosed about possible negative effects of therapy, and how such information might be divulged, is challenging. Nonetheless, the very complexities of these challenges do not practitioners from a duty to engage in thorny ethical questions. Clause 6 of the BACP's list of commitments to clients, states that practitioners must “demonstrate accountability and candor” by “being willing to discuss with clients openly and honestly any known risks involved in the work.” Evidence also indicates indicate that psychotherapy is not risk-free. However, unlike in pharmacology, there is no authority in psychotherapy or clinical psychology that obligates researchers to report on potential harms, side-effects, and unwanted events of psychological treatments (59, 60). Lilienfeld estimates that around 10% of patients experience a worsening of symptoms after receiving long-term psychotherapy (61); Crawford et al. found that 5% of UK patients reported enduring negative effects from undergoing psychotherapy (62). While in the latter case, it is unknown whether such negative effects are directly attributable to psychological treatments as opposed to other factors (such as external life events, or further deterioration of mental health), these findings underscore the importance of finding ways of communicating information about possible negative experiences in a way that empowers the client, and avoids negative effects (63–66).

### Therapeutic Benefits to Clients

Aside from the ethical duty to respect patient decision-making, participants appeared to be unfamiliar with the possible benefits of informed consent processes. It has variously been argued that informed consent may enhance levels of trust for therapists, and foster a greater sense of control and personal ownership among clients over the psychotherapy process (5, 31, 67, 68). Additional research lends support to this, suggesting that disclosing information, as well as the ongoing elicitation of client feedback, can strengthen the therapist-client alliance; improve psychotherapy outcomes; and prevent early drop-out (69–71).

### The “Hidden Ethics Curriculum”

Finally, a major theme was the problem of “ethics training by osmosis”—that is, the role of the “hidden curriculum” in psychotherapy education—a term that is more familiar in medical education (25). In our study, unintended lessons communicated to trainees are likely to have reinforced omissions, oversights, and a general laxity about securing ethical informed consent. Some students described inconsistencies with informed consent and contractual protocols during work placements, and a few expressed concerns about witnessing coercion in therapy. Equally, and despite prior awareness that therapy was mandatory in their MA course, a number of trainees felt that treatment, including aspects of it, were “*forced*.” These comments might indicate inadequate modeling by supervisors or personal psychotherapy on placements. Perhaps illustrative of a problematic hidden ethics curriculum, trainees had scarce recall of informed consent procedures as clients in therapy.

## Strengths and Limitations

A major strength of this study is the inclusion—for the first time—of trainees' voices in discussion about psychotherapy ethics. Focus groups allowed us to develop rich insights into conceptual and procedural understanding, as well as psychotherapy ethics education at a leading British university with a BACP accredited course. While health researchers are conflicted about the optimal number of focus groups, with some suggesting that one focus group is enough to obtain valuable information, it would have been useful to have continued enrolment until thematic saturation had been obtained recruitment constraints precluded this possibility. Participants in each of the two groups (second year and third year trainees) may have had subtly different understandings and experiences of informed consent as a result of their length of training. Although there was homogeneity in responses among groups, the small sample size limited our ability to discern any important differences between students' understanding and attitudes about informed consent in each year group. All students in the study were female, and whilst the majority of trainees on the course, and in the profession, are female, it is possible that participant gender may have affected the findings. We suggest that soliciting the views of male trainee therapists on informed consent would also be a fruitful avenue for future research.

The role of focus group moderators should be “non-threatening” and “supportive” (72) participants' familiarity with the research students may have influenced responses. In addition, the decision to participate in focus groups may have been influenced by students' level of engagement in the topic which, in turn, may have affected responses. Participants appeared candid in voicing their experiences and perceived misgivings about aspects of their course experiences, however, recruits may have been apprehensive about communicating other criticisms or challenges associated with their training program which may have undermined the validity of the findings.

Notwithstanding these limitations, these focus group findings provide a foundational exploration of psychotherapy trainees understanding of ethics, as they are about to embark on professional practice. Given the important emergent themes from the data set, further empirical research will help to clarify our findings. Quantitative surveys would help to assess the extent to which these results are widespread in UK among psychotherapy students; and in-depth qualitative interviews would allow for finer-grained analysis of psychotherapy trainees' understanding and opinions. Beyond research on psychotherapy students' views, we recommend that additional qualitative research focus on the views of psychotherapy and counseling educators, and psychotherapists in full-time practice. Finally, we urge that, when it comes to assessing the impact of current disclosure practices, much greater attention should be given to understanding the opinions and needs of psychotherapy clients.

## Conclusions and Implications

This preliminary descriptive analysis provides insights into the ways in which psychotherapy trainees understand and implement informed consent. Participants' comments revealed a broad variety of interpretations about, and attitudes to, informed consent. While some trainees displayed a grasp of the importance of disclosing adequate information to clients, many participants in this study were skeptical of the duty to secure ethical informed consent, or were confused about how such processes could be successfully enacted. A number of comments indicated a significant disconnect between formal psychotherapy ethics education, and obligations in practice settings. Such omissions and oversights may have been deepened by deleterious features of the hidden ethics curriculum—including students' experiences in psychotherapy as clients, and on work placements.

As we consider these findings, we cannot help but query whether formal ethics curricula and education, as well as practical aspects of psychotherapy training, are suitably equipping trainees for their ethical obligations toward clients—a concern that was noted by some participants. While we observe that psychotherapy ethics remains a relatively under-investigated field, we emphasize that this lends urgency to the need to carefully assess, and rethink, conventional wisdom to advance best ethical practices with respect to informed consent. Notably, students' views stand in opposition to the perspectives of many psychotherapy ethicists and practitioners who have recently urged the need for renewed thinking about standards of good practice to ensure respect for client autonomy. We suggest that psychotherapy and clinical psychology organizations such as the BACP and the APA should provide greater clarity to practitioners and educators when it comes to professional expectations about informed consent, including with respect to such challenging issues such as disclosures about: the nature of psychotherapy including accessible information about techniques, available treatment options, and information about common factors; advice on how consent should work as an ongoing process; and the specific implications of evidence-based practice for ethical informed consent.

As in other aspects of psychotherapy practice, progress is possible: ethical guidelines evolve, and standards of practice are refined. We hope that the results of this preliminary survey prompt timely questions about how psychotherapy curricula and training might better equip future psychotherapists to fulfill their obligations to provide ethical and effective client care.

## Data Availability Statement

The datasets generated for this study are available on request to the corresponding author.

## Ethics Statement

The studies involving human participants were reviewed and approved by the University of Leeds. The patients/participants provided their written informed consent to participate in this study.

## Author Contributions

Conceived the research: CB, TA, and GP. Conducted the focus groups and transcribed the focus groups: TA. Analyzed the focus groups: CB, JK, and CL. Wrote the first draft of paper: CB. Revised the manuscript: CB, CL, JK, TA, GP, TK, and JG.

### Conflict of Interest

The authors declare that the research was conducted in the absence of any commercial or financial relationships that could be construed as a potential conflict of interest.
